# A gut microbiome metabolite paradoxically depresses contractile function while activating mitochondrial respiration

**DOI:** 10.1242/dmm.049975

**Published:** 2023-05-15

**Authors:** Saba Naghipour, Joshua J. Fisher, Anthony V. Perkins, Jason N. Peart, John P. Headrick, Eugene F. Du Toit

**Affiliations:** ^1^ School of Pharmacy and Medical Science, Griffith University, Southport, QLD 4215, Australia; ^2^ School of Medicine and Public Health, The University of Newcastle, Callaghan, NSW 2308, Australia

**Keywords:** Cardiovascular disease, Contractility, Coronary flow, Mitochondrial respiration, Trimethylamine-N-oxide

## Abstract

Trimethylamine-N-oxide (TMAO) is an end-product of gut microbiome metabolism linked to cardiovascular disease (CVD). However, precise cardiovascular influences of the TMAO concentrations reported in early or severe disease remain to be detailed. We investigated acute effects of TMAO on cardiac contractile, coronary and mitochondrial function. Male C57Bl/6 mouse hearts were Langendorff perfused to assess concentration-dependent effects of TMAO (1-300 µM) on left ventricular (LV) function, coronary flow and select protein expression. Effects of 10 µM and 100 µM TMAO on LV mitochondrial function were examined via respirometry. TMAO at 10-300 μM concentration-dependently depressed LV contractile function, with coronary flow paralleling changes in isovolumic pressure development. Direct coronary effects were evident at >30 µM TMAO in hearts performing minimal isovolumic work, although this response was reduced by >65%. In contrast, exposure to 10 µM or 100 μM TMAO increased mitochondrial complex I, II and maximal respiratory fluxes while appearing to reduce outer membrane integrity. Expression of phosphorylated AMPKα and total GSK-3β declined. Thus, acute exposure of mouse hearts to TMAO levels reported in advanced CVD significantly inhibits cardiac contractility and induces modest coronary constriction while paradoxically overactivating mitochondrial respiration.

## Teaser

**Summary:** Acute exposure of mouse hearts to trimethylamine-N-oxide levels reported in human disease differentially depresses cardiac contractile function while inappropriately activating mitochondrial respiration at Complexes I and II.

## INTRODUCTION

The gut microbiome and associated metabolites are now understood to link our diets to a range of modern chronic diseases, including cardiovascular disease (CVD) (Blum, 2017), type 2 diabetes mellitus (Li et al., 2020) and major depressive disorder (Limbana et al., 2020). Nonetheless, we do not fully understand the mechanisms by which the gut biome influences disease development. The bacteria-dependent metabolite trimethylamine-N-oxide (TMAO), generated primarily from dietary animal protein, may participate mechanistically in these linkages, for example by contributing to the development of atherosclerosis and ischaemic heart disease (IHD) (Wang et al., 2011). However, its roles in the aetiology of CVD remain to be defined and are somewhat controversial (Naghipour et al., 2021). High dietary intakes of animal products rich in carnitine (Koeth et al., 2013), choline (Wang et al., 2011) and betaine (Naumann et al., 1983) can saturate small intestine transport processes (Sheard and Zeisel, 1986; Sanford and Smyth, 1971), directing these substrates to the colon, where select bacteria may utilise them in generating trimethylamine (TMA) (Al-Waiz et al., 1992). This precursor can then be oxidised by hepatic flavin monooxygenases (FMOs; primarily the FMO3 subtype) to produce TMAO (Lang et al., 1998). In sufficient levels, this gut-derived metabolite may influence multiple disease processes, including inflammation (Seldin et al., 2016), oxidative stress (Sun et al., 2016) and atherogenesis (Zhou et al., 2022).

Elevations in circulating TMAO are observed with increasing age (Li et al., 2017) and obesity (Sun et al., 2017), together with chronic diseases that include atherosclerosis and IHD (Cabout et al., 2017; Ding et al., 2018), diabetes (Shan et al., 2017), heart failure (HF; Tang et al., 2014) and chronic kidney disease (CKD; Tang et al., 2015). Although the presence of extracellular TMAO is consistent with potential involvement in disease, whether it plays a causal role in these disorders or represents a concomitant biomarker remains unclear (Naghipour et al., 2021). Significant disparities exist between circulating concentrations in healthy and diseased cohorts and those necessary to induce pathological effects in *in vitro* and *in vivo* models (Naghipour et al., 2021). Importantly, studies investigating the effects of TMAO on the heart often employ exceedingly high TMAO concentrations and yield diverse and sometimes conflicting results (Organ et al., 2016; Savi et al., 2018; Makrecka-Kuka et al., 2017). Moreover, very few studies have examined the effects of TMAO on cardiac mitochondrial function (Makrecka-Kuka et al., 2017; Videja et al., 2020). Although inhibitory effects on myocyte contraction, relaxation and Ca^2+^ dynamics have been reported with 20 µM TMAO (Savi et al., 2018), these responses appeared concentration independent over the limited range tested. Others report disruption of murine cardiomyocyte T-tubule organisation and Ca^2+^ handling with as little as 0.3 μM TMAO (Jin et al., 2020), despite this concentration being up to an order of magnitude lower than that observed in healthy animals (Chen et al., 2017a). Conversely, extremely high, supra-physiological TMAO concentrations (0.3-3 mM) reportedly improve cardiac inotropy (Oakley et al., 2020). Studies have indicated that concentrations as high as 10 mM do not influence cardiomyocyte viability, sarcomere length, intracellular reactive oxygen species (ROS) or mitochondrial membrane potential (Querio et al., 2019). In contrast, others have reported that 20 µM TMAO suppresses substrate- and oxidative phosphorylation-dependent respiration (and substrate flux via pyruvate dehydrogenase) in cardiac fibres (Makrecka-Kuka et al., 2017). There is also evidence that TMAO may destabilise atrial electrophysiology to promote fibrillation (Yu et al., 2018), although the local concentrations achieved with injections of 300 nmol TMAO into atrial ganglionic plexi are unknown.

The present study characterises the acute influences of TMAO concentrations observed in healthy (≤3 µM) and disease models (10-300 µM) in the mouse heart, assessing (1) myocardial contractile function (including measures of inotropic and lusitropic state); (2) coronary flow; (3) mitochondrial respiratory function and control; and (4) myocardial respiratory complex and kinase expression.

## RESULTS

### Cardiodepressant effects of TMAO

**Fig. 1.**
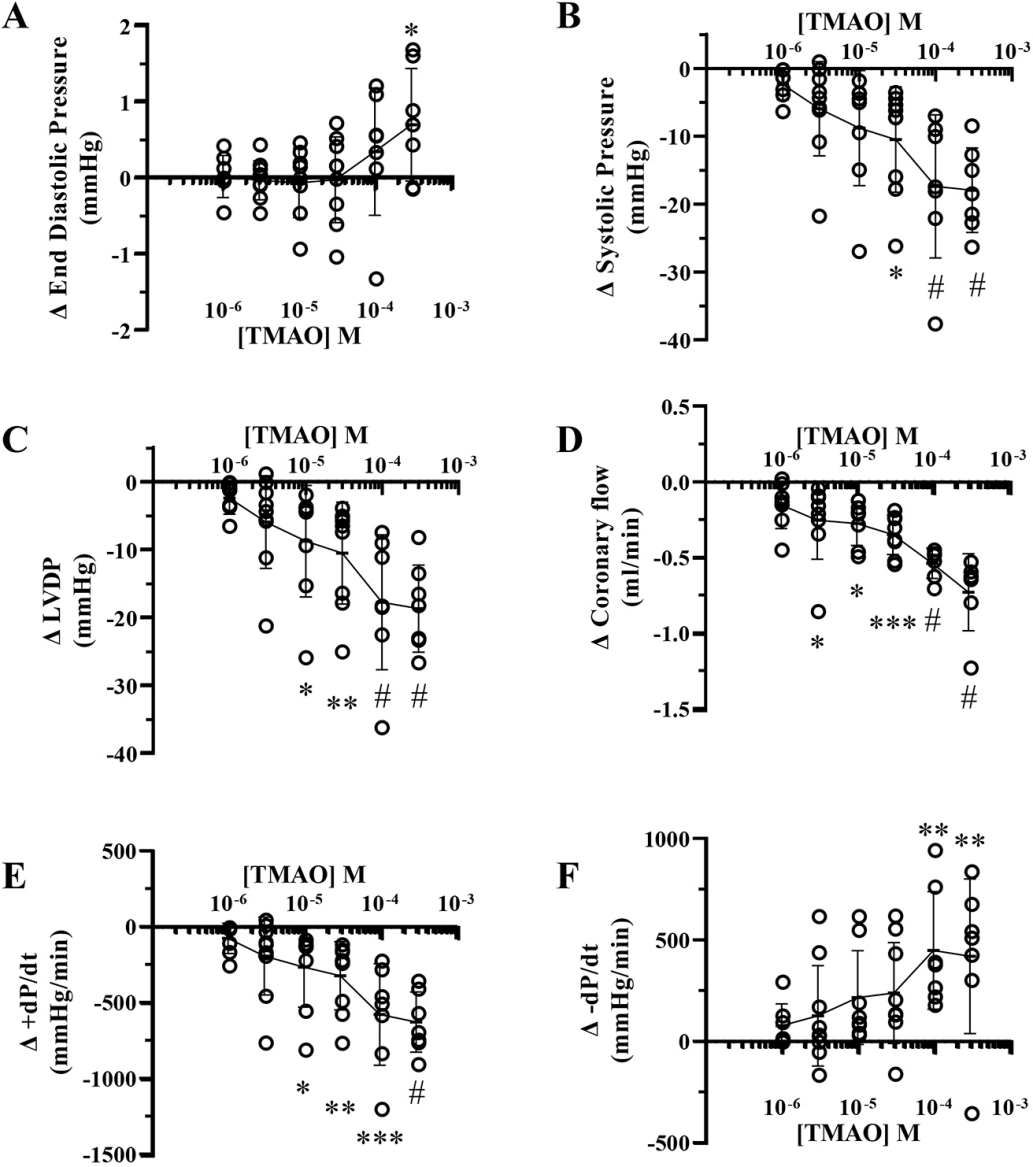
**Changes in ventricular function and coronary flow during acute trimethylamine-N-oxide (TMAO) infusion.** Data are reported as change (Δ) from baseline (pre-infusion). (A) End-diastolic pressure (*P=0.045). (B) Systolic pressure (*P=0.011; ^#^*P*<0.0001). (C) Left ventricular developed pressure (LVDP) (*P=0.047; **P=0.009; ^#^*P*<0.0001). (D) Coronary flow (*_3µM_*P*=0.022; *_10µM_*P*=0.016; ***P=0.001; ^#^*P*<0.0001). (E) Inotropic state, indicated by +d*P*/d*t* (*P=0.045; **P=0.007; ***P=0.0003; ^#^*P*<0.0001). (F) Lusitropic state, indicated by −d*P*/d*t* (**_100µM_*P*=0.003; **_300µM_*P*=0.006). Note that a positive Δ for −d*P*/d*t* reflects a reduction in this parameter. Data are presented as mean±s.d. Responses were assessed in a total of nine hearts, with five to nine TMAO concentrations tested within each heart. A one-way ANOVA followed by Dunnett's post-hoc test was applied to assess the effects of each concentration (compared to pre-infusion baseline).

Baseline contractile and flow data for perfused hearts used in this study are presented in Table 1. Values fall within ranges reported for this model, with high contractile force development and a coronary flow that is <50% of peak hyperaemic levels (i.e. high coronary reserve). Infusion of TMAO depressed LV contractile function, increasing end-diastolic pressure (EDP), and decreasing systolic pressure and +change (delta) in pressure (d*P)*/change (delta) in time (d*t*) and −d*P*/d*t*, together with coronary flow (Fig. 1). The change in LV EDP (Fig. 1A) was evident at >100 µM TMAO (significant at 300 μM, *P*<0.05). A fall in LV systolic pressure (Fig. 1B) was evident over the concentration range and achieved significance at 30 μM TMAO (*P*<0.05). Changes in LV developed pressure (LVDP) mirrored these results, with a significant decrease in LVDP at 10 µM TMAO (Fig. 1C). A decline in coronary flow with TMAO (Fig. 1D) generally paralleled LV contractile changes, and was significant at TMAO concentrations ≥3 μM (*P*<0.05). Net reductions in +d*P*/d*t* (Fig. 1E) and −d*P*/d*t* (Fig. 1F) largely mirrored those for systolic pressure and LVDP (note that a positive ‘Δ’ reflects a decline in the value of −dP/dt). Absolute values for +d*P*/d*t* and −d*P*/d*t* were significantly reduced at 10-100 µM TMAO (note that the Δ for −d*P*/d*t* reflects a reduction in this negative parameter).

**Table 1.**
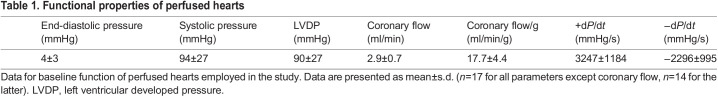
Functional properties of perfused hearts

### Influence of TMAO on coronary flow

**Fig. 2.**
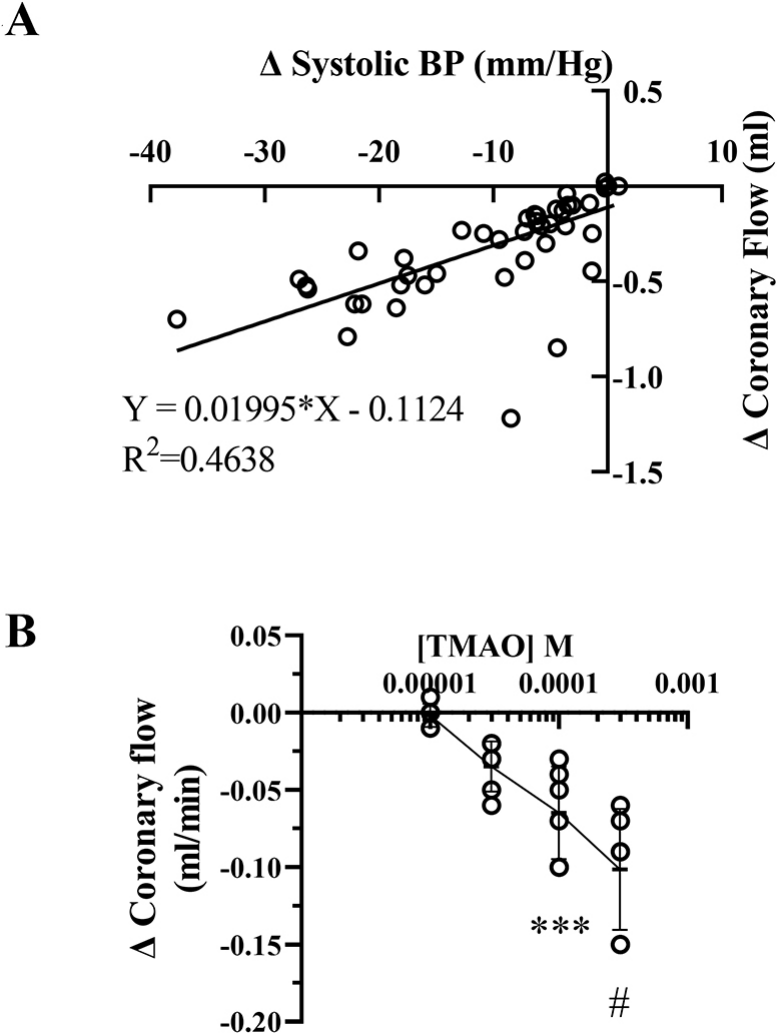
**Changes in coronary flow in hearts with or without ventricular balloons.** (A) Linear relationship between declining coronary flow and ventricular pressure development during TMAO infusion (*R*^2^=0.4638; functional data from Fig. 1; *n*=9 hearts, with five to nine TMAO concentrations tested per heart). (B) TMAO-dependent changes in coronary flow in empty hearts performing minimal isovolumic work. All data are reported as change (Δ) from baseline (***P=0.0003; ^#^*P*<0.0001; *n*=6). Data are presented as mean±s.d. A one-way ANOVA with Dunnett's post-hoc test was applied to detect the effects of each TMAO concentration (compared to pre-infusion baseline).

The decline in coronary flow that parallels LV functional changes during TMAO infusion (Fig. 1D) may reflect a direct coronary effect and/or the influence of a decline in cardiac workload or myocardial oxygen consumption (MVO_2_) (i.e. reduced functional hyperaemia). Indeed, the change in coronary flow appears strongly dependent on change in systolic pressure (*R*^2^=0.46) (Fig. 2A). To further explore the coronary influences of TMAO, responses were subsequently assessed in hearts without LV balloons, performing minimal isovolumic pressure development (Fig. 2B). In empty hearts, TMAO induced a much smaller decrease in flow (<20% of that in hearts performing significant contractile work), and the response was only significant at 100 µM TMAO (compared to 3 µM in hearts with LV balloons) (Fig. 2B).

### Antioxidant capacity increases with acute TMAO

**Fig. 3.**
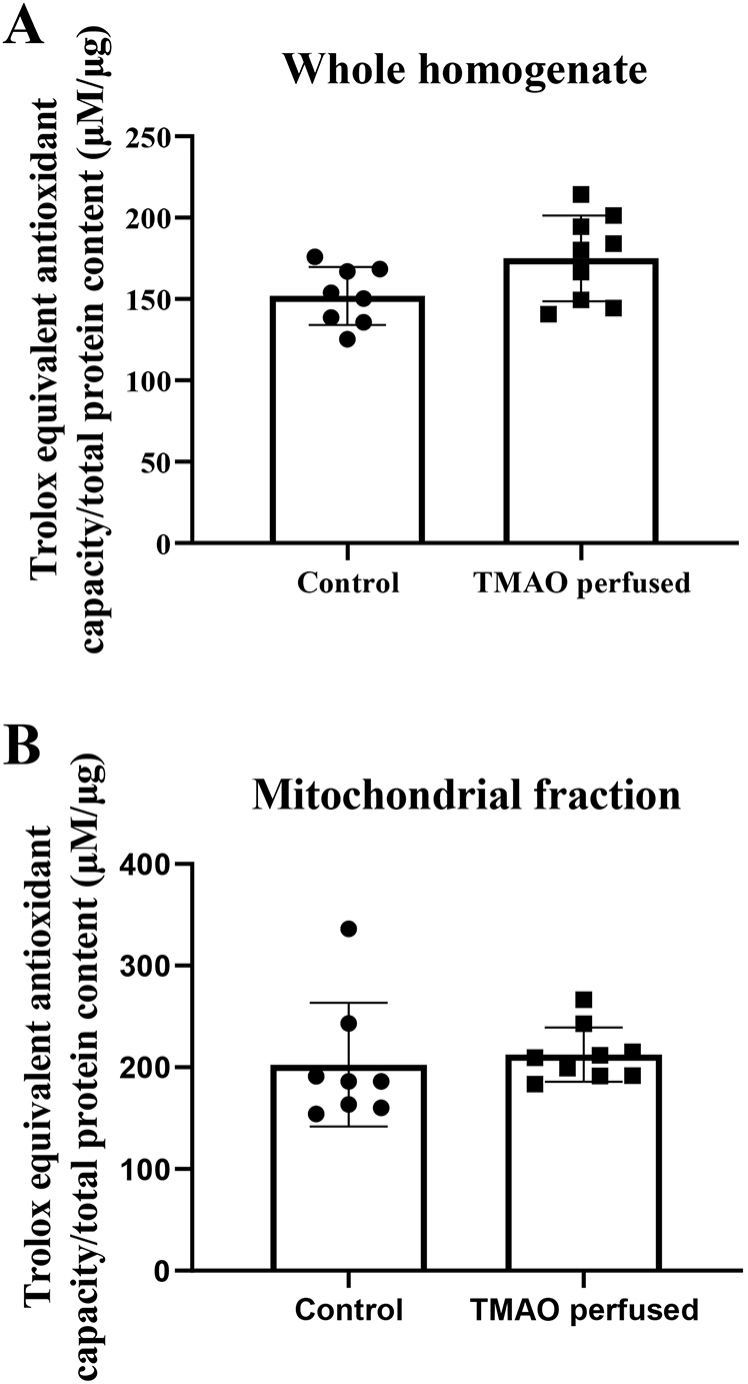
**Total antioxidant capacity in mouse hearts exposed to TMAO.** Hearts were retrogradely perfused on a Langendorff apparatus with (*n*=9) or without (*n*=8) TMAO. (A) Whole homogenate total antioxidant capacity from whole homogenised hearts. (B) Mitochondrial fraction total antioxidant capacity from whole homogenised hearts. Data are presented as mean±s.d. and were compared via unpaired two-tailed Student's *t*-test.

Total antioxidant capacity (TAC) (Fig. 3) was unaltered in either the whole homogenate (Fig. 3A) or the mitochondrial fraction (Fig. 3B) from hearts perfused with TMAO compared to control hearts.

### Effects of TMAO on mitochondrial respiration

**Fig. 4.**
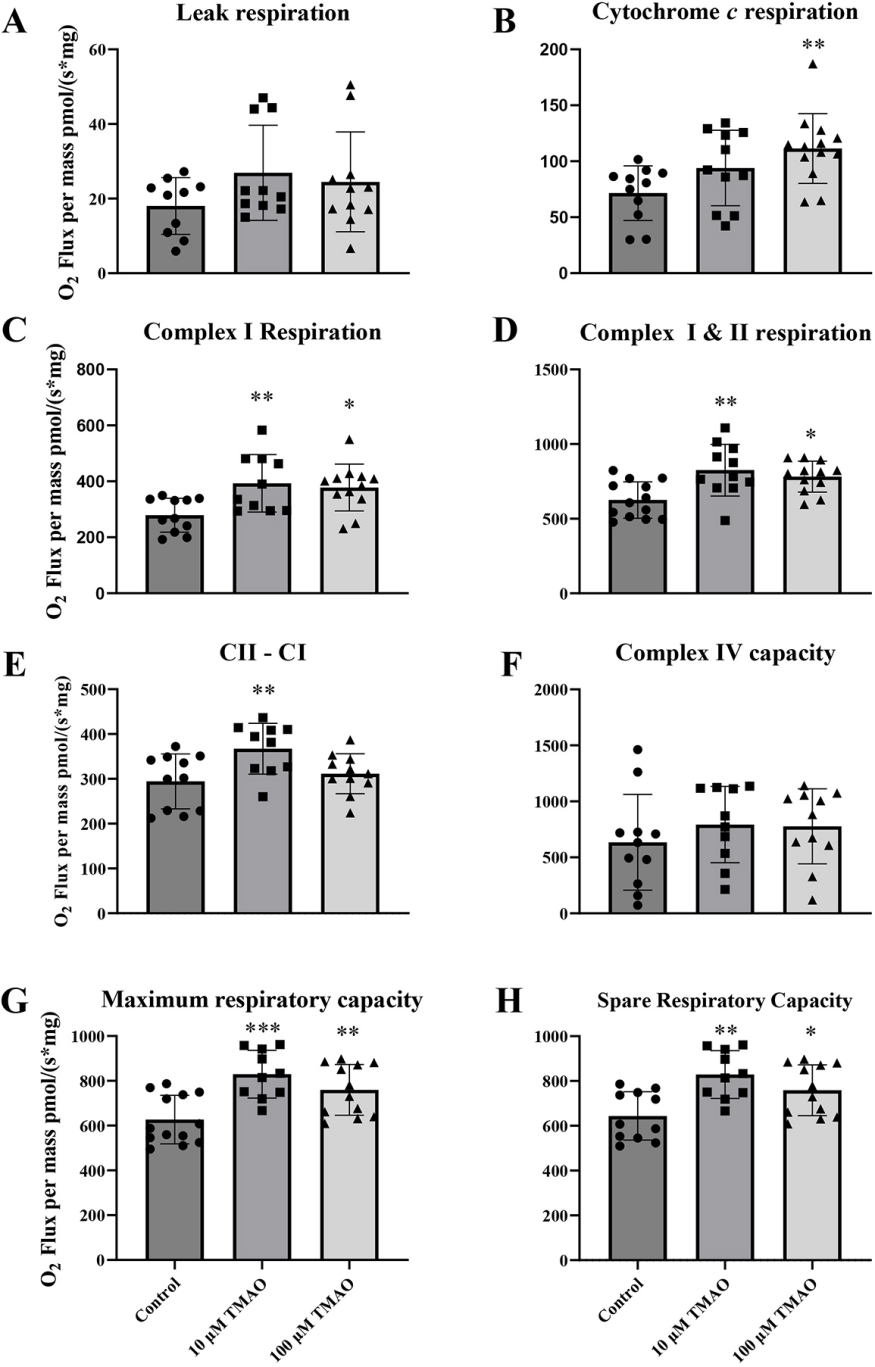
**Cardiac mitochondrial respiration under control conditions, or in the presence of 10 µM or 100 µM TMAO.** (A) Leak respiration. (B) Outer mitochondrial membrane integrity (indicated by cytochrome *c* respiration) (**P=0.005 versus control). (C) Complex I respiration (*P=0.015 versus control; **P=0.007 versus control). (D) Complex I+II respiration (*P=0.013 versus control; **P=0.002 versus control). (E) Complex II respiration (**P=0.009 versus control). (F) Complex IV capacity. (G) Maximum respiratory capacity (**P=0.009 versus control; ***P=0.0002 versus control). (H) Spare respiratory capacity (*P=0.033 versus control; **P=0.0011 versus control). Data are shown as mean±s.d. A one-way ANOVA with Dunnett's post-hoc test was applied to identify the effects of TMAO (versus control). Control, *n*=13 hearts; 10 µM TMAO, *n*=11 hearts; 100 µM TMAO, *n*=13 hearts.

Mitochondrial respiration was examined during acute exposure to 10 µM or 100 µM TMAO (Fig. 4). Although leak state respiration (Fig. 4A) was unchanged across all groups, there was a trend to increase with 10 µM TMAO (*P*=0.17). The integrity of the outer mitochondrial membrane (OMM), assessed from the respiratory response to exogenous cytochrome *c* (Fig. 4B), appeared to be significantly disrupted by 100 µM TMAO (*P*<0.01). Complex I-linked (Fig. 4C) and complex I+II-linked (Fig. 4D) respiration were both increased with 10 µM and 100 µM TMAO, while complex II-I respiration (Fig. 4E) was increased with 10 μM TMAO (*P*<0.01) but not 100 μM TMAO. Complex IV capacity (Fig. 4F) was unchanged across all groups. Maximum respiratory capacity (Fig. 4G) was significantly increased with 10 µM and 100 μM TMAO. Spare respiratory capacity was also significantly higher with 10 µM (*P*<0.005) and 100 µM (*P*<0.05) TMAO treatment (Fig. 4H).

**Fig. 5.**
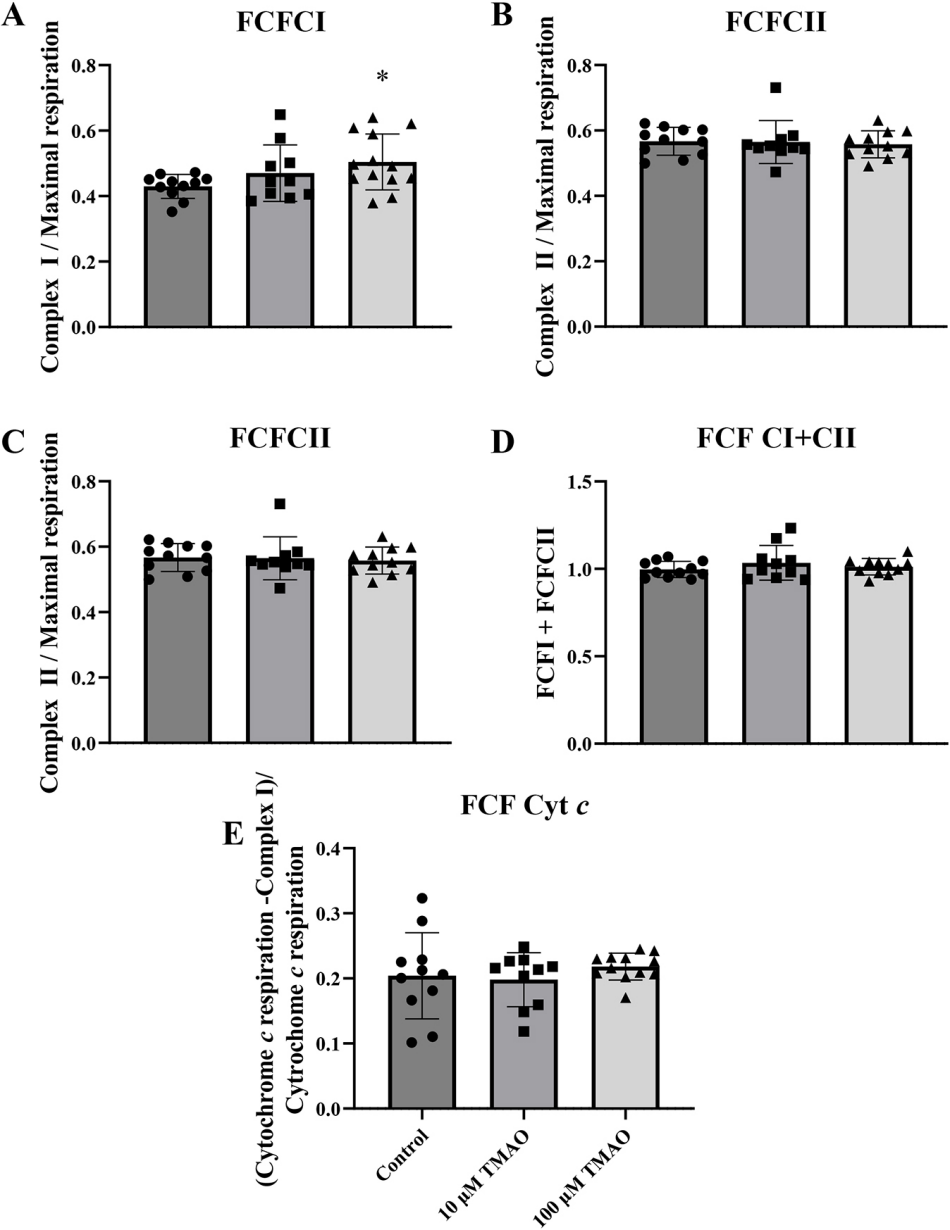
**Cardiac mitochondrial flux control ratios (FCRs) calculated under control conditions, or in the presence of 10 µM or 100 µM TMAO.** (A) Complex I (*P*<0.036). (B) Complex II. (C) Leak respiration. (D) Complex I+II-linked respiration. (E) Cytochrome *c* control efficiency. Data are presented as mean±s.d. A one-way ANOVA with Dunnett's post-hoc test was applied to identify the effects of TMAO (versus control). Control, *n*=13 hearts); 10 µM TMAO, *n*=11 hearts; 100 µM TMAO, *n*=13 hearts.

Mitochondrial respiration in different states can also be expressed as flux control ratios (FCRs) by normalising data to peak flux as a common reference state (Lemieux et al., 2011) (Fig. 5). The FCR for complex I (Fig. 5A) was increased with 100 μM TMAO (*P*<0.05) but not 10 μM TMAO. However, remaining FCR values for complex II (Fig. 5B), leak respiration (Fig. 5C) and complex I- and complex II-linked respiration (Fig. 5D) were all unchanged by TMAO. The cytochrome *c* control efficiency (Fig. 5E) was also unchanged.

Of note, there was considerable variance in several mitochondrial flux measures (particularly for leak state respiration), with a number of outlier data points identified via Grubbs' test and removed from further analysis. We included an additional more conservative analysis (see Figs S3 and S4), in which the entire datasets for each of these hearts were removed (rather than individual outlier values). The same conclusions and differences were identified with either approach.

### Cardiac protein expression

**Fig. 6.**
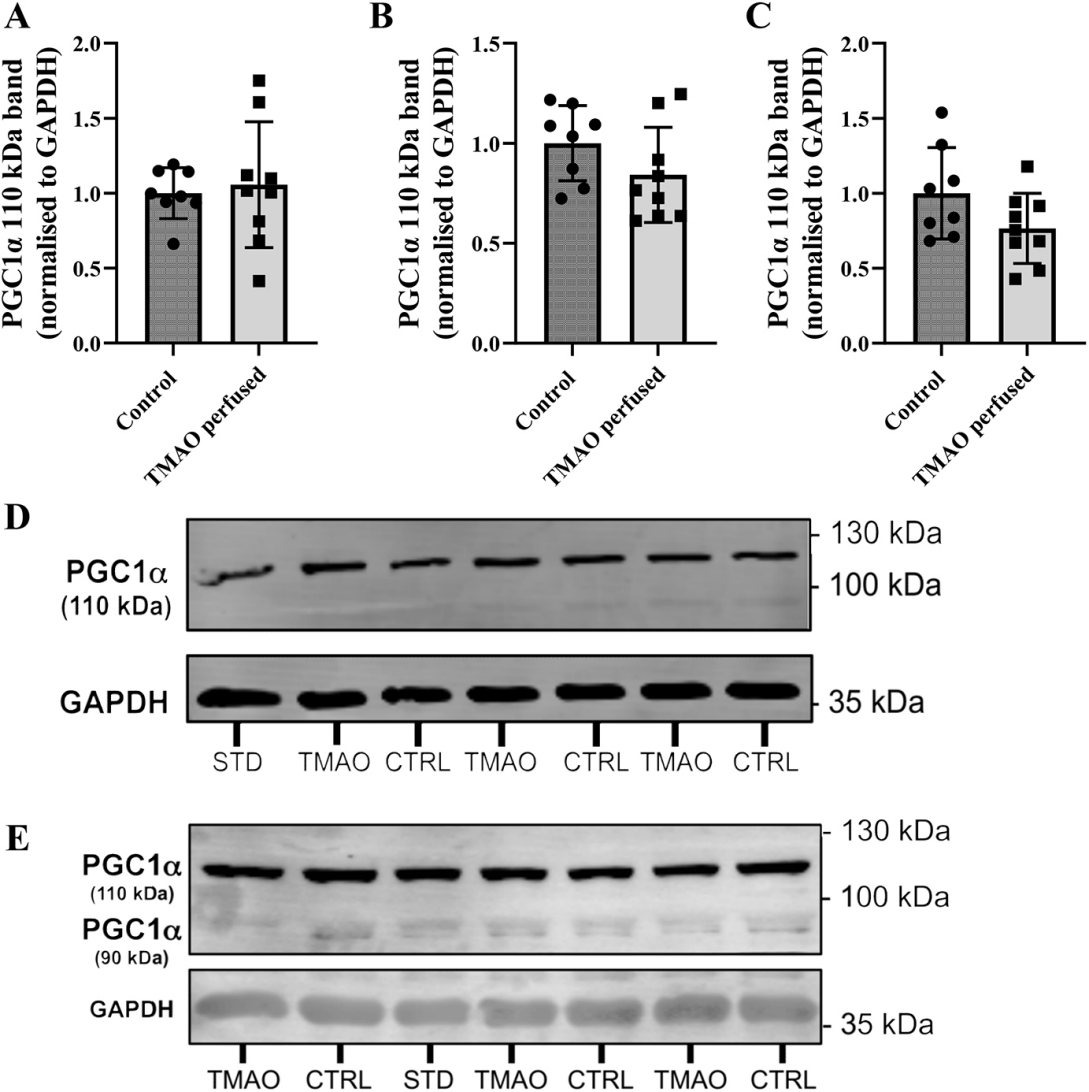
**Left ventricular expression of PGC1α in whole homogenate and cytosol-enriched fraction from hearts exposed to 1-300 µM TMAO.** (A) PGC1α (110 kDa) band in whole homogenate. (B) PGC1α (110 kDa) band in the cytosolic fraction. (C) PGC1α (90 kDa) band in the cytosolic fraction. (D) Representative blots from whole homogenate. (E) Representative blots from the cytosolic fraction. Protein expression is normalised to GAPDH and expressed relative to the control group. Data are presented as mean±s.d. (*n*=8 for control and *n*=9 for TMAO-treated hearts) and were compared via unpaired two-tailed Student's *t*-test. Lane labelling: CTRL, control hearts; STD, internal standard; TMAO, TMAO-perfused hearts.

Myocardial protein expression was assessed in whole homogenate and cytosol-enriched fractions from hearts exposed to 1-300 µM TMAO. Peroxisome proliferator-activated receptor gamma coactivator 1-alpha (PGC1α; also known as PPARGC1A) may appear at two band sizes (90 kDa and 110 kDa): we observed both bands in the cytosolic fraction but only the 110 kDa band in whole-cell homogenate (Fig. 6). Expression levels for the 110 kDa band remained unchanged in whole-cell homogenate (Fig. 6A) and the cytosol-enriched fraction (Fig. 6B), as did levels of the 90 kDa band in the cytosolic fraction (Fig. 6C). Representative blots for both are also shown (Fig. 6D,E).

**Fig. 7.**
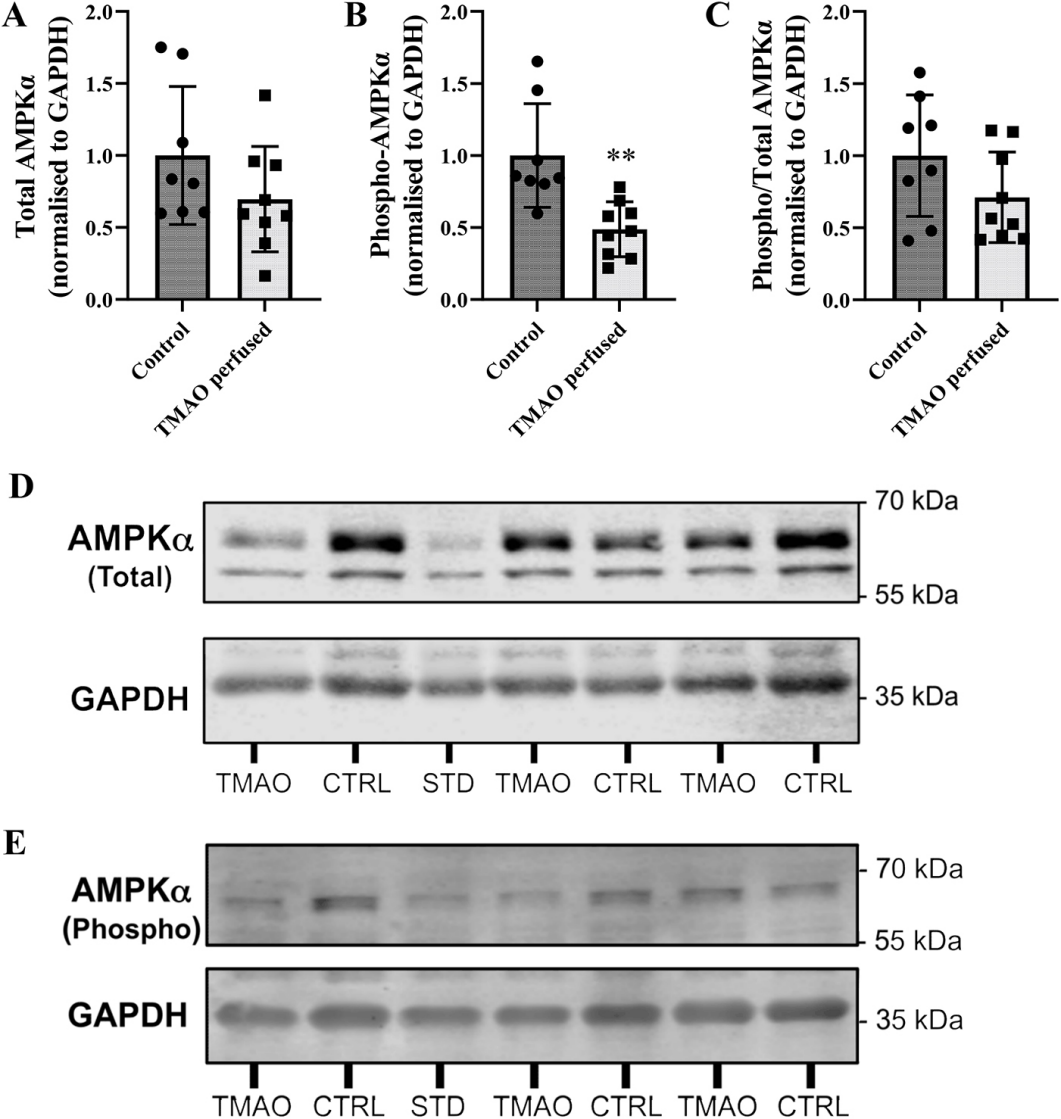
**Left ventricular expression of total and phospho-AMPKα in the cytosol-enriched fraction from hearts exposed to 1-300 µM TMAO.** (A) Total AMPKα. (B) Phospho-AMPKα (**P=0.002). (C) The ratio of phosphorylated:total AMPKα. (D,E) Representative blots. Protein expression is normalised to GAPDH and expressed relative to the control group. Data are presented as mean±s.d. (*n*=8 for control and *n*=9 for TMAO-treated hearts) and were compared via unpaired two-tailed Student's *t*-test. Lane labelling: CTRL, control hearts; STD, internal standard; TMAO, TMAO-perfused hearts.

**Fig. 8.**
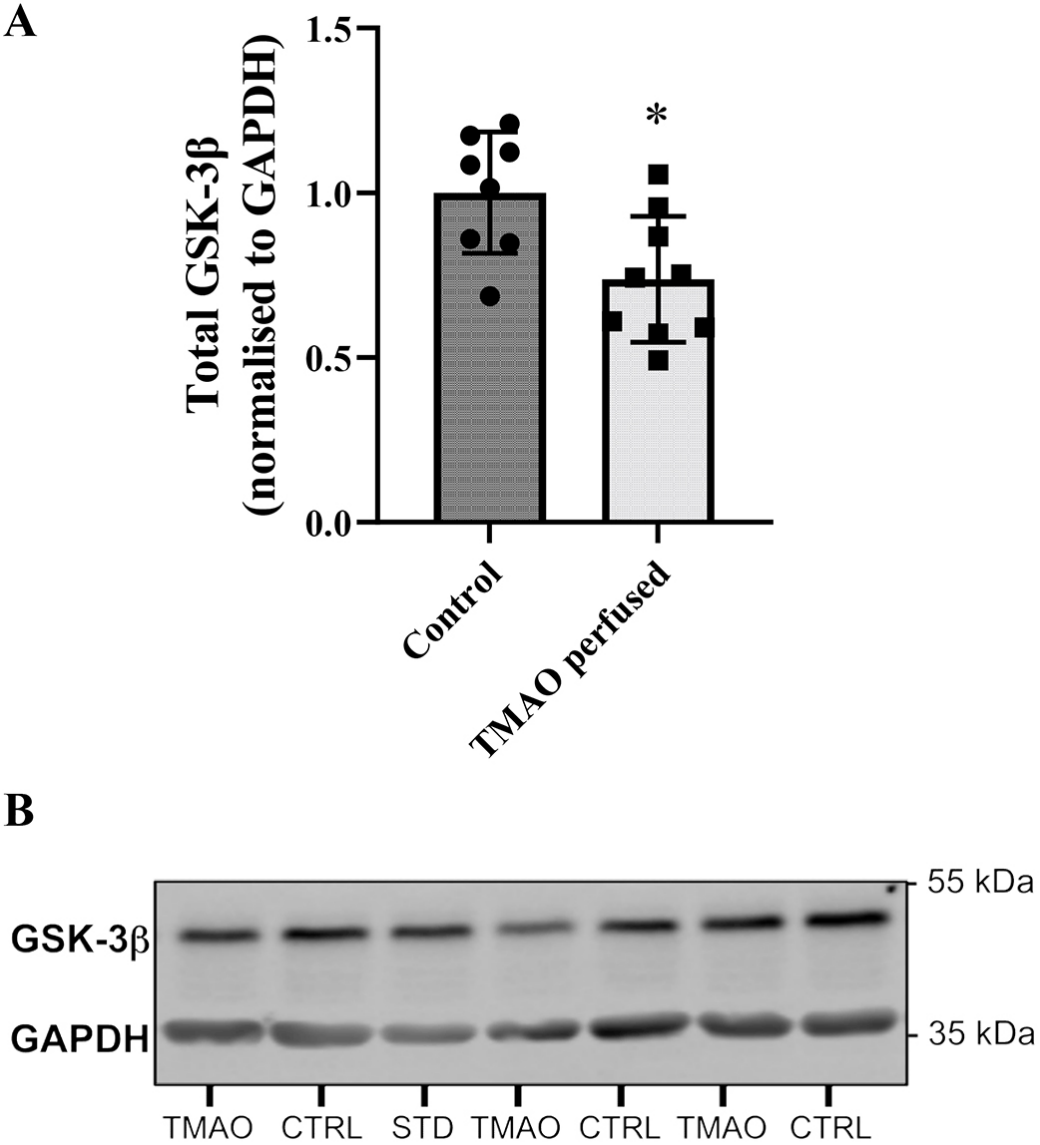
**Left ventricular expression of total GSK-3β in the cytosol-enriched fraction from hearts exposed to 1-300 µM TMAO.** (A) Total GSK-3β (*P=0.012). (B) Representative blots. Protein expression is normalised to GAPDH and expressed relative to the control group. Data are presented as mean±s.d. (*n*=8 for control and *n*=9 for TMAO-treated hearts) and were compared via unpaired two-tailed Student's *t*-test. Lane labelling: CTRL, control hearts; STD, internal standard; TMAO, TMAO-perfused hearts.

Cytosolic levels of 5′ AMP-activated protein kinase (AMPK)α were not significantly altered (*P*=0.16), despite a downward trend (Fig. 7A). However, phosphorylated (phospho-)AMPKα was significantly reduced (*P*<0.005) in TMAO-perfused hearts (Fig. 7B). As a result of these collective profiles, the ratio of phosphorylated:total AMPKα was unchanged between groups (Fig. 7C). Data are accompanied by representative blots (Fig. 7). Total glycogen synthase kinase-3β (GSK-3β) expression (Fig. 8) in the cytosolic fraction was significantly lower in TMAO-perfused hearts than in control hearts (*P*<0.05).

**Fig. 9.**
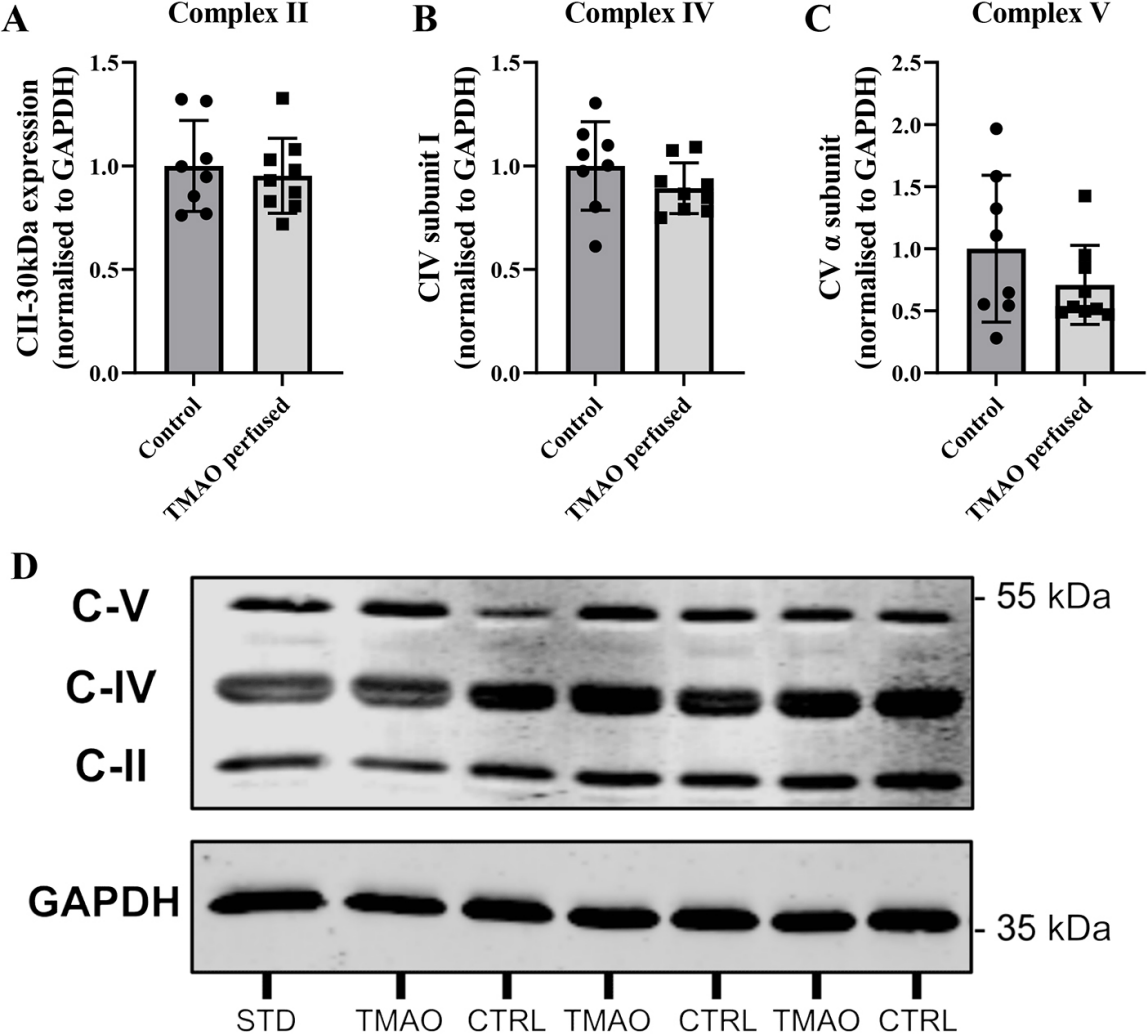
**Left ventricular expression of mitochondrial electron transport chain proteins in whole homogenate from hearts exposed to 1-300 µM TMAO.** (A) Respiratory complex II. (B) Complex IV. (C) Complex V. (D) Representative immunoblots. Protein expression is normalised to GAPDH and expressed relative to the control group. Data are presented as mean±s.d. (*n*=8 for control and *n*=9 for TMAO-treated hearts) and were compared via unpaired two-tailed Student's *t*-test. Lane labelling: CTRL, control hearts; STD, internal standard; TMAO, TMAO-perfused hearts.

An ‘oxphos’ cocktail was used to test for potential changes in cardiac expression of mitochondrial electron transport chain (ETC) complexes II, IV and V subunits (Fig. 9). The levels of these proteins in whole-cell homogenates were not influenced by TMAO exposure. No subunits we assessed were changed between groups.

## DISCUSSION

The metabolite TMAO is implicated in a number of chronic cardiometabolic disorders; however, whether relevant concentrations can impact function or energy metabolism in the heart is unclear (Naghipour et al., 2021). We tested whether acute exposure to pathophysiologically relevant concentrations (≤300 µM) of TMAO influence contractile or coronary function and cardiac mitochondrial respiration. The concentrations studied are 10- to 100-fold higher than those in healthy mice (Cabout et al., 2017), and equivalent to those reported in advanced atherosclerosis (Wang et al., 2015), IHD (Wang et al., 2011) or HF/CKD (Cabout et al., 2017) in humans. Data show that TMAO depresses contractile and coronary function in murine hearts at a threshold concentration of ∼10 µM. Somewhat paradoxically, despite this cardiodepressant effect, acute exposure to ≥10 µM TMAO increased cardiac mitochondrial respiration via complexes I and II, while potentially disrupting OMM integrity.

### Heart function

Infusion of ≥10 µM TMAO acutely decreased LV contractile function, including systolic pressure development and both +d*P*/d*t* and −d*P*/d*t*. EDP was also modestly elevated at higher TMAO concentrations. Lower (1-3 μM) TMAO concentrations reported in healthy humans (Wang et al., 2014) had no significant effects on cardiac function. Contrasting these observations, Querio et al. (2019) found that TMAO did not impact cardiomyocyte viability, ROS production, mitochondrial membrane potential or sarcomere length at very high (0.1-10 mM) concentrations. However, it has been reported that exposure to 50 µM TMAO for 24 h or 72 h reduces H9c2 cardiomyoblast viability (Lama et al., 2020), albeit when combined with abnormally high (44 mM) glucose. We also found that acute (1-2 h) incubation with 1-300 μM TMAO does not influence cellular viability in a rat cardiomyoblast line (Fig. S1, Supplementary Materials and Methods). Others described no changes in LV ejection fraction (Li et al., 2019a) in mice supplemented with TMAO in drinking water (120 mg/kg) for 8 weeks, although circulating TMAO concentration was unreported. Such a dosage regime may increase TMAO to between 15-22 µM (Makrecka-Kuka et al., 2017) and 75 µM (Videja et al., 2020). There is other evidence of an inverse correlation between plasma TMAO and LV ejection fraction (Chen et al., 2017a). Further analysis of the concentration-dependent effects of TMAO on the heart is needed, in both healthy and disease models. This is highlighted by a recent report that up to ∼75 µM TMAO preserves, rather than depresses, cardiac function in a model of right ventricular failure (Videja et al., 2020), with no apparent impact in healthy hearts.

The present data also show that TMAO does not modify myocardial antioxidant capacity. Although the TAC assay is a relatively crude measure of antioxidant function (Rubio et al., 2016), the absence of any effect on this measure is interesting. Several studies demonstrate pro-oxidant effects of chronic TMAO exposure, although within vascular elements (Li et al., 2017; Sun et al., 2016; Li et al., 2018; Ke et al., 2018). Cardiac fibrosis, linked to oxidative stress, may also be exacerbated by TMAO *in vivo* and *in vitro* (Li et al., 2019a). Nonetheless, the present data suggest little to no influence of acute TMAO on myocardial antioxidant capacity.

Coronary vascular changes during TMAO infusion were strongly dependent upon associated ventricular pressure changes, suggesting an indirect effect via shifts in functional hyperaemia. This is consistent with the much smaller (<35%) and less sensitive (100-300 μM threshold TMAO concentration) response in empty hearts performing minimal isovolumic work. Thus, high concentrations (>30 µM) of TMAO appear to have limited direct coronary effects. Prior studies indicate that very high TMAO concentrations can impair endothelial nitric oxide synthase (eNOS) and promote vascular remodelling (Sun et al., 2016). Interestingly, TMAO infusion to achieve a circulating concentration of 58 μM in rats did not modify blood pressure, but prolonged the hypertensive effect of angiotensin II (Ufnal et al., 2014), hinting at a secondary role in vascular dysfunction (Naghipour et al., 2021). One study surprisingly suggests that as little as a doubling of circulating TMAO is solely responsible for the well-documented effects of ageing on vascular function, inflammation and eNOS expression (Li et al., 2017). Similarly, vascular dysfunction in a model of CKD (associated with extremely high TMAO concentrations) is reportedly eliminated by inhibiting TMAO formation (Li et al., 2018). Further studies are warranted to validate such observations, and clarify the importance and cardiac effects of TMAO in models of disease and ageing.

### Mitochondrial respiration

Few studies have examined the cardiac mitochondrial influences of acute or chronically elevated TMAO. In contrast to a report that 20 µM TMAO decreases substrate- and oxidative phosphorylation-dependent respiration (Makrecka-Kuka et al., 2017), we found that acute exposure to 10 μM or 100 μM TMAO increased cardiac mitochondrial respiration via complexes I and II, while potentially disrupting OMM integrity. Interestingly, other work indicated that a chronic elevation in circulating TMAO to ∼75 μM decreases mitochondrial respiration while preserving it in failing hearts (Videja et al., 2020). Reasons for these diverse outcomes are unclear. Although speculative, the current data suggest that acute exposure to TMAO may induce an inappropriate respiratory activation or ‘overdrive’ in the face of depressed cardiac function (thus ATP demand). The increase in mitochondrial respiration and spare respiratory capacity in TMAO-exposed myocardium strengthens this argument. Because chronic exposure to TMAO can promote ROS generation (Sun et al., 2016; Chen et al., 2017b), it is possible that this excess metabolic activity facilitates oxidative stress. Apparent disruption of OMM integrity could be attributable to such stress, and participate in respiratory dysfunction. Previous work suggests that ‘acute’ incubation with 50 μM or 100 μM TMAO decreases pyruvate dehydrogenase activity in isolated cardiac mitochondria (Makrecka-Kuka et al., 2017). Conversely, the present study supports an acute TMAO-dependent increase in pyruvate-driven respiration, although pyruvate dehydrogenase activity is not directly assessed here. Among other factors, differing outcomes may be attributable in part to sample preparation: mitochondrial respiration in homogenised myocardial tissue versus that in a mitochondria-enriched fraction. Because other cellular components may consume oxygen, this could modify respiratory measures. However, respiratory functional data are normalised to residual oxygen consumption (achieved after inhibition of complexes I and III), and additional analysis revealed that this parameter is unchanged between treatment groups (Fig. S2).

### Protein expression

Evidence is acquired for significant post-translational influences of TMAO, including reduced levels of phospho-AMPKα and total GSK-3β expression. However, ETC component expression was unchanged. Both PGC1α (Navarro et al., 2017) and AMPK (Hardie, 2007) influence mitochondrial biogenesis, increasing PGC1α activity via increased protein expression and phosphorylation (Jäger et al., 2007). In the present study, cardiac PGC1α expression was not influenced by TMAO. It has also been reported that PGC1α mRNA in rat cardiomyoblasts is unaltered by 10 µM TMAO (Huang et al., 2020), although the duration of treatment was not stated. One study demonstrates that inhibiting TMA (hence TMAO) production appears to lower PGC1α mRNA, albeit in skeletal muscle (Schugar et al., 2022). Given the lack of change in PGC1α expression, we did not further study additional targets (e.g. nuclear respiratory factor 1/2, transcriptional mitochondrial factor A).

Although total AMPKα was unchanged, phospho-AMPKα was significantly reduced by TMAO. Activation of AMPK involves the phosphorylation of Thr172 in the α-subunit, and AMP and/or ADP binding to the γ-subunit (Hardie et al., 2012), and occurs during periods of stress or de-energisation: an increase in the AMP/ATP ratio (reflecting de-energisation) favours AMPK activation (Hardie, 2007). Increased ADP-driven mitochondrial respiration with TMAO might be relevant to the fall in phospho-AMPKα, because phosphorylation state declines with increasing energy state (Jeon, 2016). Studies assessing TMAO effects on AMPK are few: work done in vascular smooth muscle and endothelial cells revealed that TMAO (1 mM) treatment for 24 h decreases total AMPK expression (Zhou et al., 2022), although phosphorylation was not assessed or discussed, and the amount of TMAO used is considered supra-physiological (Naghipour et al., 2021). Further investigating this phosphorylation change, we additionally assessed GSK-3β, which not only influences mitochondrial function and dependent cell death pathways, but influences cardiac function (Michael et al., 2004), and can interact with AMPK and phosphorylate the α-subunit at the Thr172 site (Suzuki et al., 2013). The TMAO-dependent reduction in GSK-3β expression observed here could contribute to the shift in AMPKα phosphorylation.

Lack of substantial changes in respiratory protein expression suggests that the acute influences of TMAO on mitochondrial respiration are largely post-translational in nature. This is consistent with the relatively brief period of TMAO treatment (∼1 h). Future studies might compare the effects of chronic versus acute TMAO exposure on ETC expression and functionality. To the best of our knowledge, only one other study has investigated the effects of TMAO on ETC protein expression, reporting a fall in COX1 (presented as complex IV) after 2 weeks’ exposure to TMAO (Yoshida et al., 2022).

### Limitations and future directions

Although studies here detail the functional and mitochondrial influences of TMAO, the mechanistic basis of these responses remains to be detailed. Nonetheless, data provide clues as to candidate mechanisms. For example, the apparently inappropriate activation of respiration and OMM disruption with TMAO may promote ROS generation, further influencing mitochondrial and myofibrillar elements and excitation–contraction coupling. Increased ROS levels interfere with myocyte Ca^2+^ current and channels (Guerra et al., 1996) and promote arrhythmogenesis (Goldhaber, 1996). An increase in EDP evident with high TMAO (300 μM) is consistent with abnormal Ca^2+^ handling. We also show that TMAO can influence the expression and phosphorylation state of AMPKα, offering a potential post-translational basis for TMAO effects on contractility and/or respiration. In addition to pursuing such mechanisms, future studies could also investigate the effects of long-term elevations in TMAO, rather than the acute exposure assessed here. Such changes in exposure time might allow for additional transcriptional or translational influences of the metabolite.

Finally, although it is most relevant to assess the cardiovascular influences of extracellular TMAO released from the liver, it would be of interest to identify intracellular concentrations achieved within cardiomyocytes (e.g. at the level of myofibrils or mitochondria) and other cell types. The trans-membrane concentration gradients for TMAO in different cells are largely unknown, although it is noteworthy in this regard that similar TMAO concentrations influence metabolic function in both intact tissue and isolated mitochondria (Makrecka-Kuka et al., 2017).

### Conclusions

The studies described here demonstrate that acute exposure to ≥10 µM TMAO can moderately depress contractile function while inappropriately increasing mitochondrial respiration at complexes I and II in murine hearts. The extracellular concentrations inducing these effects are 10- to 100-fold higher than those reported in healthy mice, and equivalent to concentrations reported in murine models of atherosclerosis (Koeth et al., 2013), HF (Li et al., 2019b) and CKD (Li et al., 2018), and in human CKD (Cabout et al., 2017) and advanced IHD (Wang et al., 2011). Relevance to human disease nonetheless awaits clarification, with studies in human cardiovascular tissue needed. Chronicity of exposure is likely to be an important factor worthy of future investigation: long-term exposure to even lower levels of TMAO may be detrimental to the heart. The current data are consistent with roles for TMAO in the later progression of cardiac disease and dysfunction, rather than in the early genesis of disease. The molecular bases for these contractile and mitochondrial responses await further investigation.

## MATERIALS AND METHODS

### Ethics and animals

All investigations were approved in accordance with policy guidelines (The Animal Care and Protection Act 2001) of the Animal Ethics Committee of Griffith University (ethics approval MSC/04/19/AEC), which is accredited by the Queensland Government, Department of Primary Industries and Fisheries, under the guidelines of The Animal Care and Protection Act 2001, section 757, Australia. Studies were undertaken using male C57Bl/6 mice, sourced from the Animal Resource Centre (Perth, Australia) at 8 weeks of age and housed in the Griffith University Animal Facility for the duration of studies. Mice were housed in groups of four to five with sawdust bedding and *ad libitum* access to water and food, and habituated to the facility for at least 2 weeks prior to experimentation. The mice were maintained in a 12 h day–night lighting cycle at a constant temperature of 21°C and 40% humidity. Animals (12-13 weeks of age) were used in the two primary arms of the study: (1) assessment of functional responses to 1-300 µM TMAO in Langendorff perfused hearts; and (2) analysis of the influences of 10 µM and 100 µM TMAO on mitochondrial respiration in shredded LV myocardium.

### Isolated Langendorff heart model

We employed a Langendorff perfused mouse heart model supplied with glycolytic substrate, previously characterised by us and shown to generate superior contractile force at submaximal coronary flows (hearts are O_2_ sufficient and optimally functional), exhibit strong cardiac-coronary coupling and high levels of cellular energisation (Headrick et al., 2001). Male mice (12-13 weeks) were anaesthetised with a single intraperitoneal injection of 50 mg/kg sodium pentobarbitone, before undergoing a thoracotomy and removing the heart into ice-cold perfusion fluid. The aorta was cannulated, and hearts were perfused in a retrograde fashion at a hydrostatic pressure of 100 mmHg, as previously described (Reichelt et al., 2009), with modified Krebs bicarbonate buffer containing the following: 120 mM NaCl, 25 mM NaHCO_3_, 4.7 mM KCl, 1.2 mM KH_2_PO_4_, 2.5 mM CaCl_2_, 1.2 mM Mg_2_SO_4_, 15 mM glucose, 0.6 mM EDTA and 2 mM pyruvate. Perfusate was equilibrated with 95% O_2_, 5% CO_2_ at 37°C, giving a pH of 7.4. Perfusate temperature was maintained at 37°C, and hearts were constantly bathed in perfusate within a small water jacketed chamber maintained at 37°C. The left ventricle was vented with a polyethylene drain to prevent accumulation of Thebesian drainage. Coronary perfusion was constantly monitored via a cannulating ultrasonic flow-probe (Transonic Systems Inc., Ithaca, NY, USA) in the aortic perfusion line, and aortic perfusion pressure was monitored using a P23XL pressure transducer (Viggo-Spectramed, Oxnard, CA, USA) connected to a MacLab data acquisition unit (AD Instruments, Castle Hill, Australia). Aortic pressure was held constant at 100 mmHg. After an initial 20 min of stabilisation at intrinsic heart rate, all hearts were electrically paced via silver electrodes at a constant rate of 420 beats/min (Grass S9 stimulator; Grass Instrument Company, Quincy, MA, USA), in order to eliminate rate-dependent changes in contractile function. Hearts were allowed to stabilise for an additional 10 min before experimentation. Hearts (*n*=9) were exposed to incremental concentrations of TMAO (1-300 µM) in the perfusion fluid (5 min at each concentration). A second group of hearts was studied (*n*=6), in which no ventricular balloon was present, limiting the isovolumic work performed and allowing direct vascular responses to be distinguished from shifting metabolic control during changes in contractile function. Finally, a third group of untreated hearts (*n*=8) was perfused under control conditions for use as a comparator group in protein expression analyses.

### Cardiac mitochondrial function

Mice were sacrificed via cervical dislocation before the thoracotomy. This was undertaken to avoid potential mitochondrial influences of sodium pentobarbitone (Hertsens et al., 1984). Hearts were immediately rinsed in MiR05 respiration medium [0.5 mM EGTA, 3 mM MgCl_2_·6H_2_O, 60 mM K-lactobionate, 20 mM taurine, 10 mM KH_2_PO_4_, 20 mM HEPES, 110 mM sucrose, 1 g/l fatty acid-free bovine serum albumin, pH 7.1 (Krumschnabel et al., 2015)] before LV tissue (∼4-7 mg) was isolated and placed into 1 ml MiR05 respiration medium±10 µM TMAO, or 100 µM TMAO. Tissue was then shredded using an Omni Tissue Homogenizer (OMNI International, Kennesaw, GA, USA) with ten strokes over 10 s using the first gear, followed by five strokes over 5 s on the second gear. Samples were adjusted to a final concentration of 1 mg tissue/ml MiR05 respiration medium, in the absence (*n*=13) or presence of 10 µM (*n*=11) or 100 µM (*n*=12) TMAO.

Mitochondrial respiratory function was monitored (at 37°C) in a Oxygraph-2k (Oroboros, Innsbruck, Austria) using the substrate–uncoupler–inhibitor–titration (SUIT) protocol. A 2.2 ml sample of tissue homogenate was added to each chamber. Following baseline calibration (5 min), pyruvate (5 mM), malate (2 mM) and glutamate (10 mM) were added prior to titration of ADP (1-5 mM) to assess complex I leak and maximum respiration, respectively. Cytochrome *c* (10 μM) was added to assess OMM integrity, followed by succinate (10 mM) to determine complex I+II-linked maximum respiration. Treatment with carbonyl cyanide m-chlorophenyl hydrazone (CCCP; 0.05 µM) was used to measure maximum respiratory capacity. Complex I and III inhibition were achieved with rotenone (0.5 μM) and antimycin A (2.5 µM), respectively, providing measurement of residual oxygen consumption (ROX). Complex IV capacity was then assessed by sequential addition of ascorbate (2 mM) and N, N, N′, N′-tetramethyl-p-phenylenediamine (TMPD; 0.5 mM). The ROX was subtracted from all respiratory rates to eliminate non-mitochondrial respiration. To ensure that optimum conditions were maintained for oxidative phosphorylation, chamber oxygen concentrations were maintained above 30 µM by opening the chamber to the stop position allowed a gas phase to come into contact with the liquid phase until baseline concentrations (180-200 µM) were restored. All respiratory rates were recorded via Oroboros DataLab 7.0 software, and units of respiration are expressed as pmol O_2_/s/mg tissue before statistical analysis.

### Myocardial antioxidant capacity

A Trolox equivalent assay was used to measure LV oxidant capacity (total cell and mitochondrial). Then, 2,2′-azino-di-3-ethylbenzthiazoline sulphonate (ABTS) was dissolved in water to a concentration of 7 mmol/l. ABTS radical cation (ABTS•^+^) was produced by reacting the ABTS solution with 2.45 mmol/l potassium persulfate. The mixture was left in the dark at room temperature for 12-16 h before use. ABTS•^+^ stock solution was diluted to an absorbance of 0.7 at 750 nm using either PBS (pH 7.4) for the study of hydrophilic antioxidant activity, or ethanol for the study of lipophilic antioxidant activity. Whole homogenate and mitochondrial fraction samples (5 µl) were added to a 96-well flat-bottom plate in triplicate. Trolox (6-hydroxy-2,5,7,8-tetramethychroman-2-carboxylic acid) was used as a standard and dissolved in PBS or ethanol (final concentration 0-40 µM). Diluted ABTS•^+^ solution (190 µl) was added to all wells, and absorbance measurements were taken at 750 nm at 30°C, 5 min after the initial mixing. Measurements were taken using a Tecan Sunrise Absorbance Reader with Magellan Standard software (TECAN, Grödig, Austria). Decolourisation of the assay was linear with increasing concentrations of the standard, Trolox. The results were expressed relative to Trolox activity and protein content (μM Trolox equivalent).

### Myocardial protein expression

We assessed levels of different proteins known to participate in regulating mitochondrial biogenesis (PGC1α), together with energy metabolism and mitochondrial function, permeability transition pore activity and dependent pathways of cell death/apoptosis (AMPKα and GSK-3β). All three proteins are also known to influence cardiomyocyte viability and stress resistance.

#### Tissue homogenisation and fractionation

Whole hearts were snap frozen in liquid N_2_ and stored at −80°C until analysis (*n*=8-10). To enrich for subcellular fractions, whole tissue was homogenised in ice-cold mitochondrial isolation buffer: 70 mM sucrose, 190 mM mannitol, 20 mM HEPES, 0.2 mM EDTA, 1 mM PMSF, 10 µM leupeptin, 3 mM benzamidine, 5 µM pepstatin A and 1 mM NaO). A portion of whole homogenate was removed and mixed with an ice-cold lysis buffer (10 µM leupeptin, 3 mM benzamidine, 5 µM pepstatin A and 1 mM NaO, 1% Triton X-100) and stored at −80°C. To enrich for subcellular fractions, the remaining homogenate was centrifuged at 600 ***g*** for 10 min at 4°C, with the pellet (nuclear) washed in mitochondrial isolation buffer, re-spun at 600 ***g*** for 10 min at 4°C, re-suspended in lysis buffer and stored at −80°C; the supernatant (mitochondria, cytosol and plasma membrane) was re-centrifuged at 10,000 ***g*** for 30 min to obtain a mitochondrial fraction (pellet) that was washed in mitochondrial isolation buffer, re-spun at 600 ***g*** for 10 min at 4°C, re-suspended in lysis buffer and stored at −80°C. The remaining supernatant (cytosol and plasma membrane) was spun at 100,000 ***g*** for 1.5 h to obtain a cytosolic fraction (supernatant) and a plasma membrane fraction (pellet), the latter of which was re-suspended in lysis buffer and stored at −80°C. The supernatant enriched for cytosol was transferred to a new tube and stored at −80°C. Protein content was determined via a Pierce^TM^ BCA Protein assay (Thermo Fisher Scientific; Scoresby, Australia), and aliquots containing 30 µg protein were prepared.

#### Western blotting

Once thawed, protein aliquots were prepared and denatured with equal volumes of loading dye. A 30 μl volume of sample was loaded into hand-cast 10% or 12% (for the rodent ‘oxphos cocktail’) acrylamide gels. Protein separation was achieved by running gels at 50 V for 10 min, followed by 120 V for 60-90 min. Transfer of proteins was achieved using a polyvinylidene difluoride membrane at a constant 75 V for 2-2.5 h, followed by a wash with Tris-buffered saline (TBS) for 10 min, blocking with Odyssey fish serum for an additional 1-1.5 h at room temperature and a final 10 min TBS wash. Transferred proteins were incubated with primary antibodies overnight at 4°C with gentle agitation: total oxphos rodent cocktail (1:500, mouse polyclonal, ab110413, Abcam); anti-PGC1α (1:1000, rabbit polyclonal, ab72230, Abcam), anti-AMPKα (1:1000, rabbit polyclonal, s2532, Cell Signaling Technology), anti-phospho-AMPKα (1:1000, rabbit polyclonal, s2531, Cell Signaling Technology), anti-GSK-3β (1:1000, rabbit monoclonal, 9315, Cell Signaling Technology) and anti-GAPDH (1:500, mouse monoclonal, sc-32233, Santa Cruz Biotechnology; 1:500, rabbit monoclonal, 2118, Cell Signaling Technology).

The membranes were washed four times in TBS mixed with Tween 20 (TBST; 5 min each) and again in TBS (10 min) before a 1 h incubation with the corresponding secondary antibodies at room temperature in the dark: IRDye^®^ 680RD donkey anti-mouse (1:30,000, 925-68072, LI-COR Biosciences, Lincoln, NE, USA) or IRDye^®^ 680RD goat anti-rabbit (1:20,000, 925-68071, LI-COR Biosciences). Membranes were then re-washed four times in TBST and once in TBS (5 min and 10 min each, respectively), before drying overnight between Kimwipes and paper towels in the dark. Membranes were visualised on an Odyssey Infrared Imaging System (LI-COR Biosciences), and protein densitometry data for each sample were normalised to GAPDH (found to be consistent between groups). A common internal standard, prepared from a mix of multiple cardiac homogenate samples, was run as a positive control (and facilitated normalisation where groups or samples span multiple gels).

### Statistical analysis

All data are expressed as mean±s.d. A Dunnett's multiple comparisons test was employed in testing differences between TMAO-treated and -untreated values (corrected *P*<0.05 was considered evidence of significance). For differences between two groups, an unpaired two-tailed Student's *t*-test was used (*P*<0.05 was considered evidence of significance). A simple linear regression was performed to assess the relationship between the change in systolic pressure against the change in coronary flow. Outlier data points were removed from analysis after identification via a Grubbs’ test. This was more prominent with mitochondrial flux analyses, and two modes of outlier removal and analysis have been included in this regard (see ‘Effects of TMAO on mitochondrial respiration’ section in the Results and added analysis within Figs S3 and S4).

## Supplementary Material

10.1242/dmm.049975_sup1 Supplementary information
