# Supplementary material for: A gut microbiome metabolite paradoxically depresses contractile function while activating mitochondrial respiration
Source: Dis Model Mech. 2023 May 15;16(5):dmm049975. doi: 10.1242/dmm.049975 (PMC10214852; doi:10.1242/dmm.049975)
Supplement: Supplementary information [file dmm-16-049975-s1.pdf]

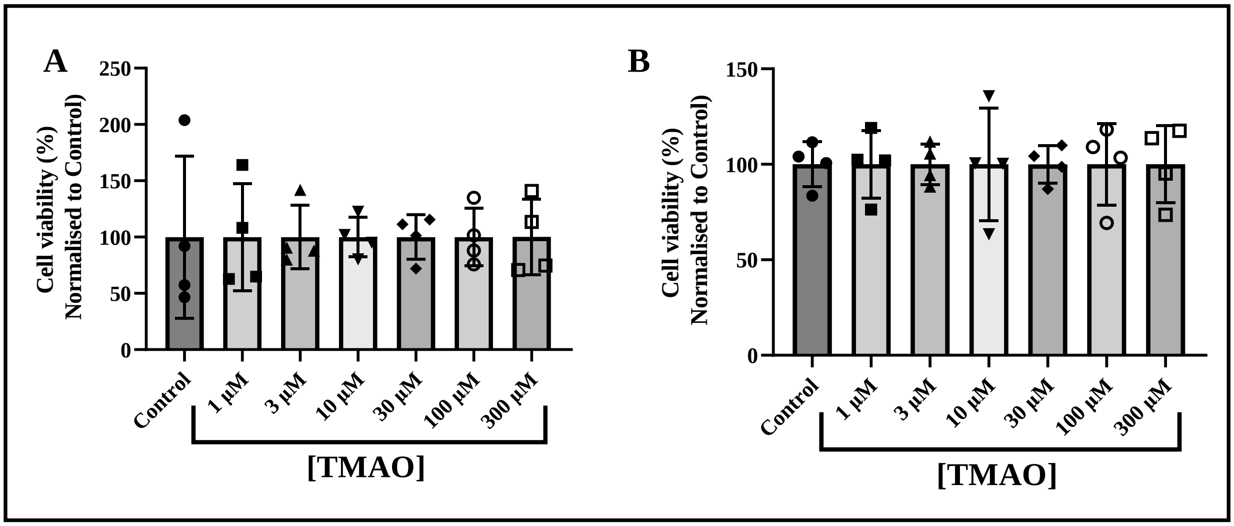

Fig. S1. Cellular viability in H9c2 cells using an MTT assay. Cells were exposed to matching concentrations of TMAO from the perfusion study for either 1 hr (**A**) or 2 hr **(B**) before measuring changes in viability. Data is normalised to Control and expressed as means±SD. *n*=4 for all groups.

Disease Models & Mechanisms • Supplementary information

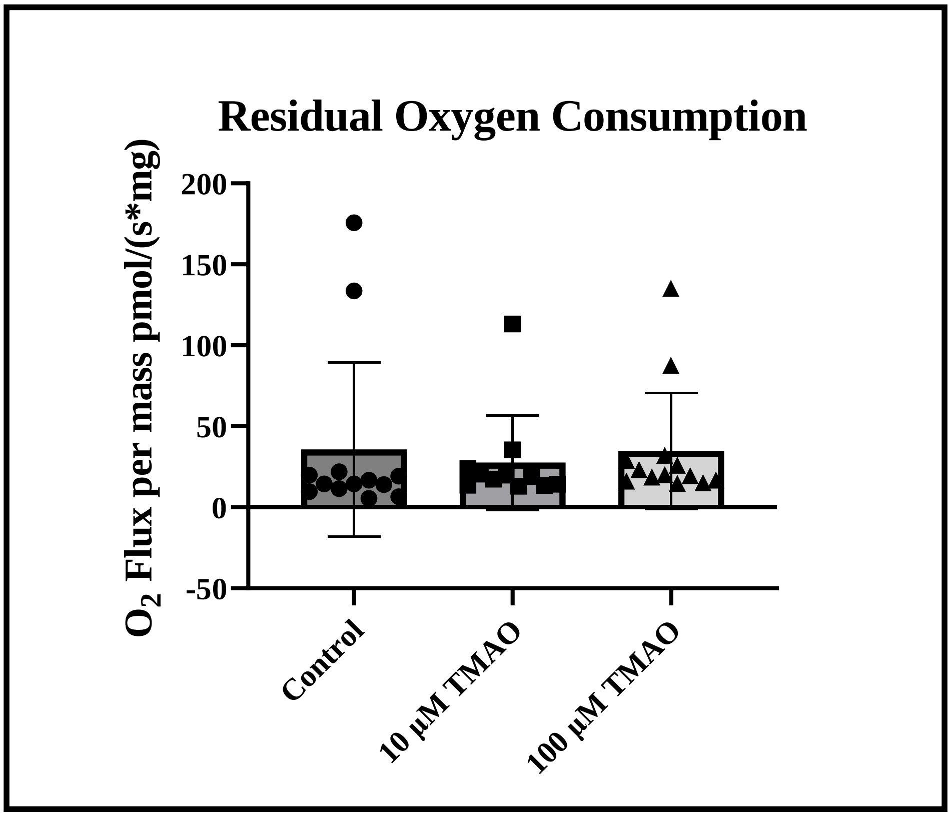

Fig. S2. Residual oxygen consumption (ROX) from hearts used in mitochondrial studies. ROX is measured after the addition of rotenone (1 µM) and antimycin A (5 mM) to inhibit Complex I and III respectively. Data is expressed as means±SD. A One-way ANOVA with a Dunnet's post-hoc test was used to compare means and no significant differences were observed between the groups. *n*=11-13.

Disease Models & Mechanisms • Supplementary information

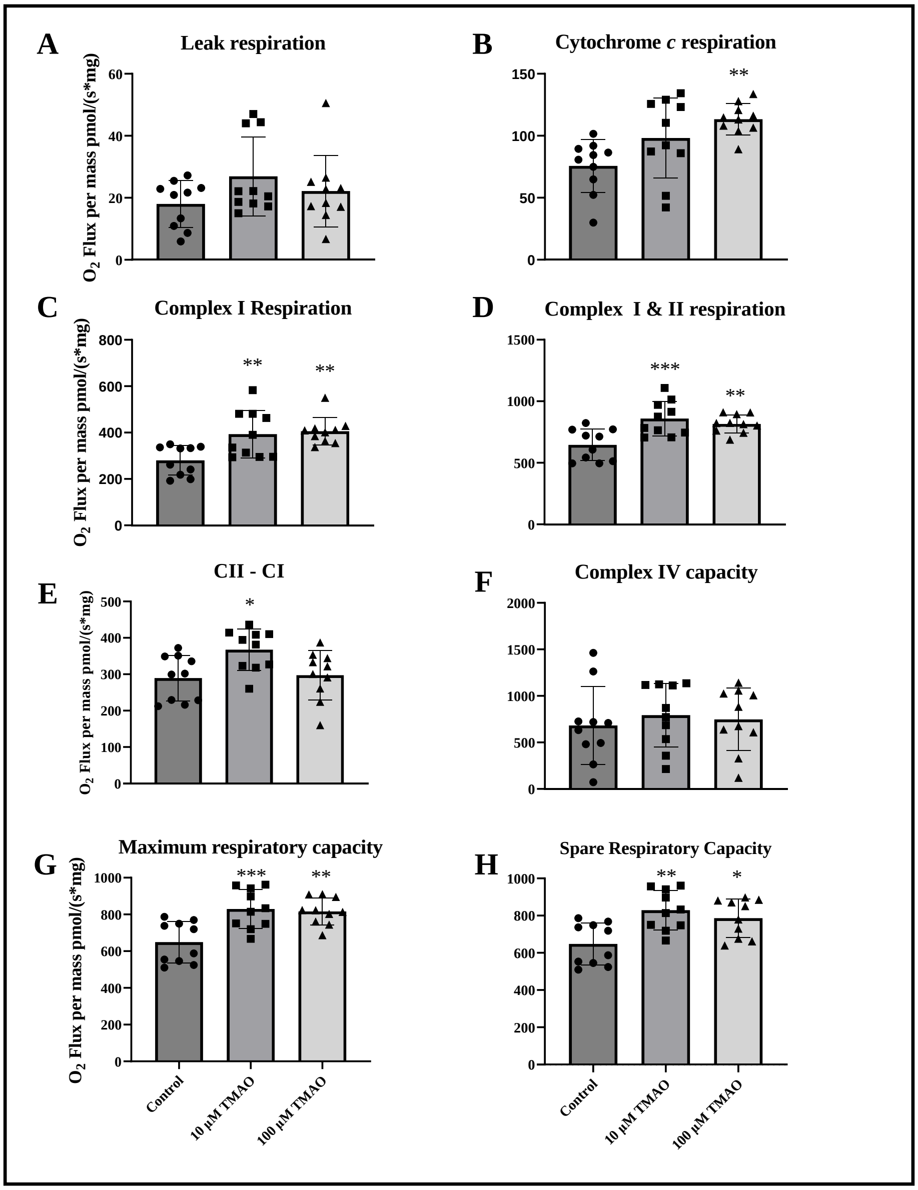

Fig. S3. Cardiac mitochondrial respiration under Control conditions (*n*=10 hearts) or in the presence of 10 µM (*n*=10 hearts) or 100 µM TMAO (*n*=10 hearts), with all data removed for every heart exhibiting one or more outlier values. Data are shown for: **A)** Leak respiration; **B)** Outer mitochondrial membrane integrity (**, *p*=0.003 *vs*. Control); **C)** Complex I respiration (**$_{10\ \mu M}$, *p*=0.006 *vs*. Control; **$_{100\ \mu M}$, *p*=0.002 *vs*. Control); **D)** Complex I & II respiration (**, *p*=0.006 *vs*. Control; ***, *p*=0.0008 *vs*. Control); **E)** Complex II respiration (**, *p*=0.01 *vs*. Control); **F)** Complex IV capacity; **G)** Maximum respiratory capacity (**, *p*=0.002 *vs*. Control; ***, *p*=0.0007 *vs*. Control); and **H)** Spare respiratory capacity (*, *p*=0.014 *vs*. Control; **, *p*=0.002 *vs*. Control). Data shown as means±SD. A one-way ANOVA with Dunnet's post-hoc test was applied to identify effects of TMAO (*vs*. Control).

Disease Models & Mechanisms • Supplementary information

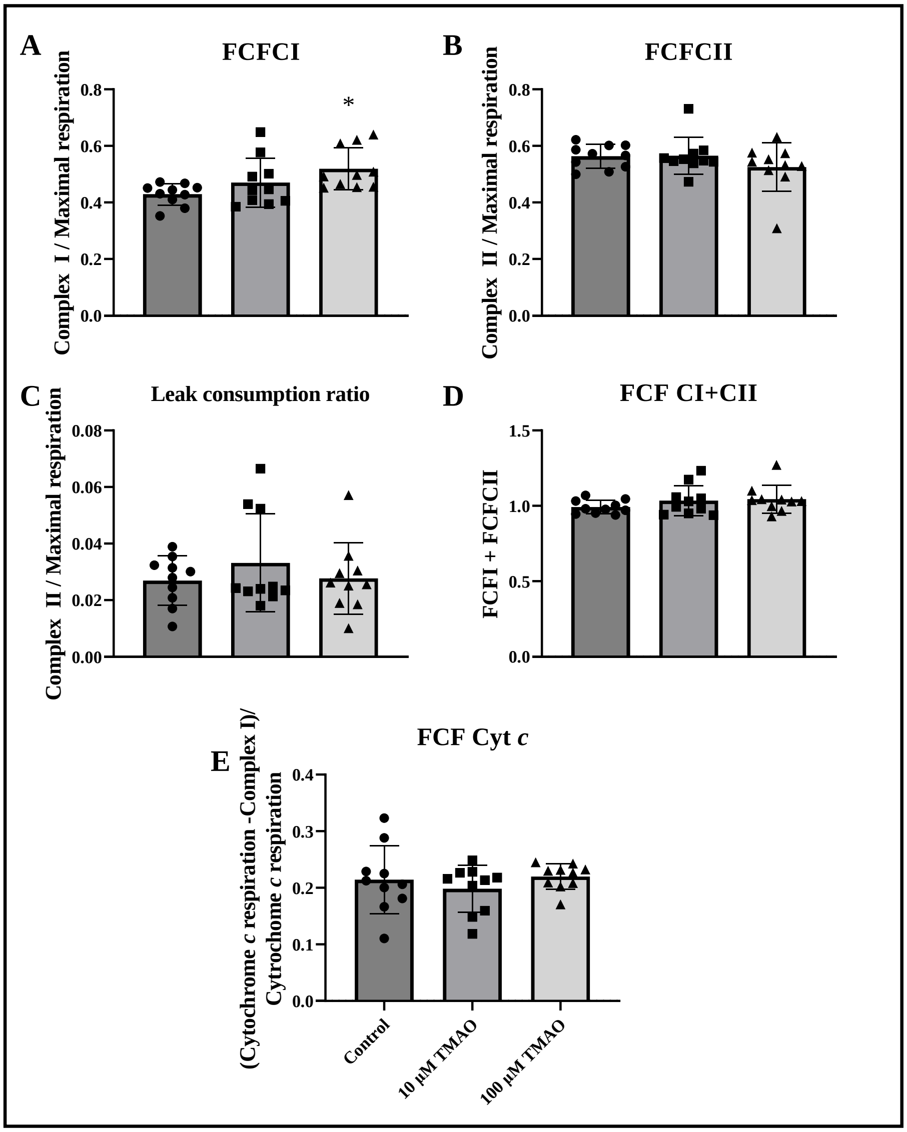

Fig. S4. Cardiac mitochondrial flux control ratios (FCRs) were calculated under Control conditions (*n*=10 hearts) or in the presence of 10 µM (*n*=10 hearts) or 100 µM TMAO (*n*=10 hearts), with all data removed for every heart exhibiting one or more outlier values. Data are shown for FCRs for: **A)** Complex I; **B)** Complex II (*, p=0.014); **C)** leak respiration; **D)** Complex I + CII linked respiration; and **E)** cytochrome *c* control efficiency. Data presented as mean±SD. A one-way ANOVA with Dunnet's post-hoc test was applied to identify effects of TMAO (*vs*. Control).

Disease Models & Mechanisms • Supplementary information

Supplementary Materials and Methods

The H9c2 rat cardiomyoblast cell line was used and purchased from Sigma-Aldrich (Castle Hill, NSW, Australia). Cells were grown and maintained in 5 mM glucose Dulbecco's modified Eagle's medium (DMEM) supplemented with 10% fetal bovine serum, 100 U/mL penicillin, and 100 µg/mL streptomycin and kept under 95% O2-5% CO2 at 37°C. Cells were used between passages 11 and 15. A 2 mM TMAO stock was diluted in DMEM to achieve the concentrations required for the study.

To assess cellular viability, a CellTiter 96 non-radioactive cell proliferation MTT assay (cat. no. G4000, Promega, Madison, WI, U.S.A) was performed. Cells were seeded into a 98-well plate at a density of 7,500 cells per well in 150 µL of 5 mM glucose DMEM and allowed to adhere for 24 hours. Media was then removed and replaced with DMEM containing TMAO to match the heart perfusion study concentrations (1 µM, 3 µM, 10 µM, 30 µM, 100 µM, 300 µM) before adding the dye solution immediately after or following one hour of treatment. A stop solution was then added to the wells 1 hour after the addition of the dye. Plates were then placed into a Tecan Sunrise Absorbance Reader with Magellan Standard software (TECAN, Austria) and absorbance was recorded at 570 nm. A reference wavelength of 650 nm was also used.
